# Effects of Size, Body Mass and Body Condition on Tonic Immobility Occurrence in *Lissotriton vulgaris*

**DOI:** 10.3390/ani16162616

**Published:** 2026-08-20

**Authors:** Simeon Lukanov, Angel Dyugmedzhiev, Miroslav Slavchev, Blagovesta Zheleva

**Affiliations:** Institute of Biodiversity and Ecosystem Research, Bulgarian Academy of Sciences, 1000 Sofia, Bulgaria; angel_diugmedjiev@abv.bg (A.D.); slmiro@abv.bg (M.S.); besta.dimitrova@gmail.com (B.Z.)

**Keywords:** antipredator behaviour, BCI, smooth newt, SVL, temperature, thanatosis

## Abstract

Tonic immobility is a state of natural paralysis that species across the animal kingdom exhibit as an instinctive response to an external threat. In ectotherms, it has been linked with temperature, but the effects of individual size, body mass and body condition are still poorly understood. We hypothesize that under natural conditions, size, body mass and body condition play an important role in tonic immobility occurrence in smaller ectothermic animals such as the Smooth newt. In this study, we photographed, measured and weighed 357 adult *Lissotriton vulgaris* from four populations across Bulgaria in the period December 2025–June 2026. All observed cases of tonic immobility were noted and subsequently analyzed against the effects of newt size, body mass and body condition, as well as ambient air temperature. Our results indicated that smaller newts, with lower body mass, were more prone to displaying the behaviour than larger newts, while sex and ambient temperature had no observable effects.

## Simple Summary

When stressed or facing an immediate threat, many animals instinctively freeze and play dead to confuse the attacker. This survival strategy is well-known and, in cold-blooded animals, is often linked to temperature. However, we still do not fully understand how individual body size, body mass, and relative body condition affect whether animals choose to use it. In this study, we examined 357 adult smooth newts from four different populations to see how their physical traits and the ambient air temperature influenced this behaviour. Our findings revealed that smaller and lighter newts were significantly more likely to play dead than larger individuals, whereas sex and temperature had no impact. This suggests that under natural conditions, physical traits and relative body condition show strong negative associations with death-feigning display probability in wild populations.

## 1. Introduction

Tonic immobility, also known as thanatosis (after the ancient Greek god of death, Thanatos), is an antipredator behaviour defined by reflex in which the individual adopts a distinct motionless posture and pretends to be dead [1]. The behaviour is displayed most often when the prey and the attacker are in physical contact, and it is widely assumed to be a “last resort” option for survival [2], although there are also reports of spontaneous occurrence of thanatosis [3].

In ectothermic animals, thanatosis has been demonstrated to be temperature-dependent [4,5]. It was first applied to amphibians in the early 1970s [6,7], and since then the behaviour has been widely reported for different taxa. Although it has mostly been studied in anuran species; e.g., [8,9,10], recently there has also been some research on caudates, e.g., [1,11]. Mounting data demonstrates that even though initially thanatosis may appear to be a simple process, it is often displayed under a wide range of situations and may include or interact synergistically with other behaviours [12]. Toledo et al. [12] also suggest that the behaviour consists of two distinct types—(1) thanatosis, or true death-feigning, and (2) shrinking, or contracting. During true death-feigning, the animal remains motionless even if touched, the eyes remain open and the limbs are loose; it is displayed when the animal is handled by the potential predator. During shrinking behaviour, the animal is again motionless, but the eyes are closed and the limbs are bent and positioned close to the body; it is displayed when the animal is approached by a potential predator, or sometimes immediately after the first physical contact [12]. It has to be noted that the literature review by Toledo et al. [12] covers only anuran species, and that 80% of the examined toxic species displayed the shrinking behaviour (only 20% displayed true thanatosis), so shrinking could serve a warning function as well.

The Smooth newt *Lissotriton vulgaris* (Linnaeus, 1758) is a small (up to 81 mm) caudate amphibian from the family Salamandridae, distributed across large parts of Western and Central Europe, the Balkan peninsula (incl. western parts of Asian Turkey) and the Caucasus, reaching as far north as Western Siberia [13]. It is widespread in Bulgaria, inhabiting small ponds, canals, puddles and other temporary lotic water bodies up to around 1800 m.a.s.l. [14]. It is also among the most terrestrial members of the genus *Lissotriton* Bell, 1839, and its body size has been shown to vary with altitude [15].

Body size and body mass are widely used in calculations of a body condition index (BCI), which serves as a primary proxy for relative energetic status and is a common approach in population ecology [16]. BCI is often used to examine the effects of different environmental factors on the animals’ fitness, either at individual or population level, and in amphibians, it reflects changes with the onset of the breeding season [17]. Cogălniceanu et al. [18] demonstrated that body size and BCI could provide reliable estimates of the impact of habitat degradation in populations of the Spadefoot toad (*Pelobates fuscus* (Laurenti, 1768)).

However, our understanding of the effects of body condition on amphibian defensive behaviour is still very limited. Body condition could directly influence the trade-off between active escape and passive immobility, as individuals with lower BCI have lower endurance, which renders active flight energetically prohibitive and functionally ineffective. Baškiera et al. [1] demonstrated that proportion of tonic immobility responses increased with body temperature in Alpine newts (*Ichthyosaura alpestris* Wolterstorff, 1934), but was temperature independent in the Smooth newt, suggesting that other factors must also play a role. In this study, we tested whether body size, body mass, or body condition have an effect on the frequency of thanatosis displays in the Smooth newt. We hypothesized that smaller, lighter newts would be more likely to display the behaviour than larger, heavier newts, or individuals with better fitness.

## 2. Materials and Methods

During the period December 2025–June 2026, we conducted monthly visits to four habitats in Western Bulgaria—a pond located near the Pastrina protected area (“Pastrina”, N43.432, E23.373, 230 m.a.s.l.), a pond located near the Parshevitsa mountain chalet (“Parshevitsa”, N43.139, 23.466, 1360 m.a.s.l.), an irrigation canal in a field near the village of Slivata (“Slivata”, N43.774, E23.048, 50 m.a.s.l.), and a pond near the village of Yarlovo (“Yarlovo”, N42.489, 23.253, 1300 m.a.s.l.). During each visit, funnel traps were set up in the evening and collected in the morning; depending on available open water area, 15–25 traps were set 3–5 m apart as to evenly cover the pond area (for the exact trap number per session and exposure time, see Table S1). All captured newts were measured (Snout-vent length (SVL); with a plastic ruler, accuracy of 0.1 cm), weighed (with a digital scale, accuracy of 0.01 g), photographed (with a smartphone; ventral pattern through a transparent plastic cover) and released at the site of capture. SVL was preferred as the structural metric over total length in order to prevent data distortion from tail damage and regeneration, while the sex-stratified models controlled for dimorphic mass allocation (e.g., male crests vs. female egg mass). Sex was determined on site based on external characteristics, and ambient air temperature was measured with a handheld thermometer (accuracy of 0.1 °C) at the time of trap collection. Displays of tonic immobility were noted and, when possible, recorded on video. All newts were handled in the same manner, without an intentional attempt to induce the behaviour. They were removed from the trap and housed together without other species in a plastic tank full of water from the respective site; newts were individually taken from the tank, photographed in the same position (manually positioned ventral side up; see Figure S1), measured and weighted; the duration of the process was 40–60 s, and after that individuals were released at the site of capture.

Photographs of the ventral pattern were used to check for recaptures between sessions. However, individual recognition could not be achieved due to the great similarity between the patterns, and repeat captures of the same individuals across sampling dates are possible. While this potential pseudoreplication represents a limitation of our sampling, monthly trapping intervals minimized the likelihood of localized re-sampling bias.

All statistical analyses were performed in R v. 4.2.1 [19], using the readxl [20], ggplot2 [21] and logistf [22] packages. For each newt, BCI score was calculated using the residuals from the regression line of ln body mass versus ln SVL, in accordance with [23], where positive scores denote individuals heavier than expected for their length and negative scores indicate lower relative energetic reserves. A single global Ordinary Least Squares linear regression, pooling all individuals across populations, was fitted to derive a standardized species-level reference baseline. Model assumptions were verified by visual inspection of residual vs. fitted value, and a Shapiro–Wilk test on the regression residuals (*p* > 0.05). Morphological divergence across study sites was evaluated independently for each sex. Differences in body mass, SVL and BCI across the four study populations were tested using one-way Analysis of Variance (ANOVA), followed by Tukey’s Honest Significant Difference (HSD) post hoc tests for pairwise site comparisons. To assess localized (i.e., specific for each site) sexual size dimorphism, a two-sample Student’s *t*-test was conducted within each population to compare males and females with regard to body mass, SVL, and BCI. To address rare-event binary bias and spatial separation across sites, Firth’s penalized bias-reduction logistic regression was implemented to model the probability of thanatosis display against individual morphometrics (body mass, SVL, BCI) and environmental context (ambient air temperature), with sex evaluated across models.

We followed Toledo et al. [12] in accepting only “true death-feigning” as a case of thanatosis, as opposed to cases of curled newts with closed eyes or a head under their body. The behaviour itself was coded as a binary outcome, i.e., a newt could either display or not display thanatosis, with no intermediate outcomes. To provide geographic context, study sites were grouped into “Lowlands” (Slivata and Pastrina) and “Highlands” (Yarlovo and Parshevitsa), and were visually mapped across behavioural responses. Statistical significance was accepted at *p* < 0.01, with values between 0.01 and 0.05 considered marginally significant. All figures were generated using the ggplot2 package.

## 3. Results

A total of 357 adult newt captures were recorded, and all animals were measured, weighed, photographed and released at the site of capture. At all sites, most newts were captured in the period March–May; only Yarlovo had captures in December, while for January, February and June there were no captures (Table S1). Displays of tonic immobility were noted and in some cases documented with a short video (Video S1).

### 3.1. Morphometrics

Mean values for SVL and body mass for both sexes from each study site are presented in Table 1. In all four populations, males were always larger, but only at one site (Pastrina) were they also heavier (Table 1).

**Figure 1:**
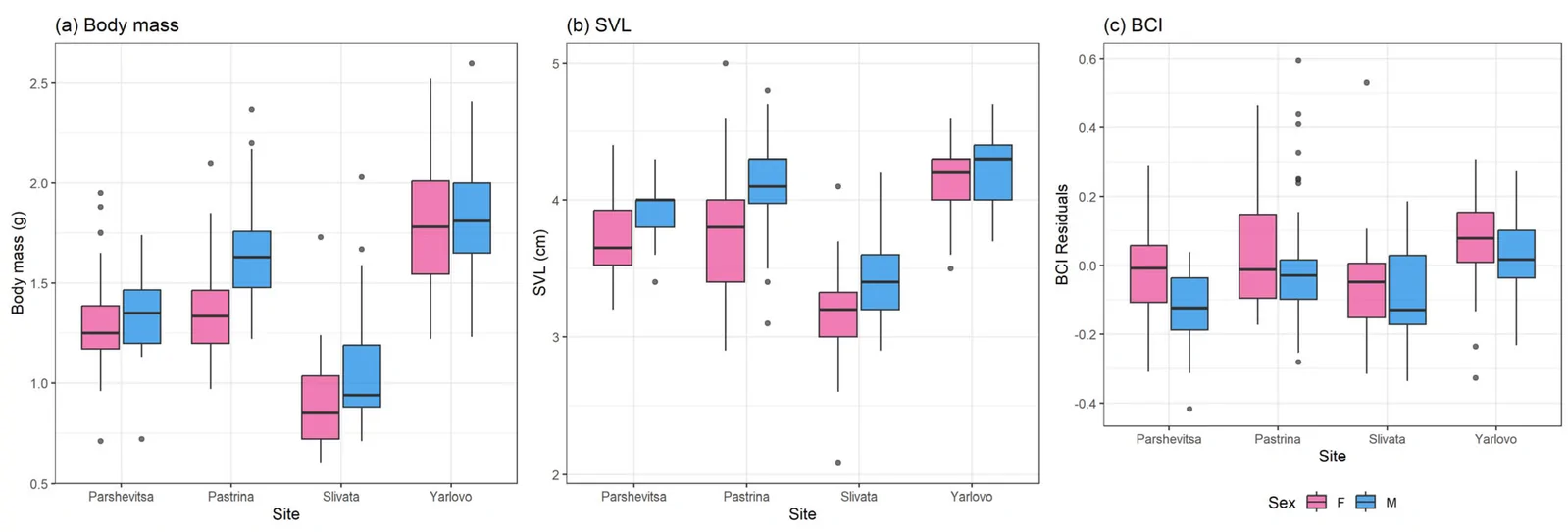
Comparison by site and sex of (**a**) Body mass, (**b**) SVL and (**c**) BCI. In each boxplot, the horizontal line represents the median, the box limits define the 25th and 75th percentiles, and solid circles denote individual statistical outliers.

For both males and females, there were statistically significant differences between the study sites in terms of body mass (df = 3, males F = 87.84, *p* < 0.001; females df = 3, F = 74.46, *p* < 0.01), SVL (males df = 3, F = 89.86, *p* < 0.001; females df = 3, F = 57.09, *p* < 0.001) and BCI (df = 3, males F = 11.38, *p* < 0.001; females df = 3, F = 6.53, *p* < 0.001). The Tukey HSD post hoc test demonstrated differences between most study sites in terms of body mass and SVL, while differences in BCI were less pronounced (Table 2, Figure 1).

### 3.2. Thanatosis

In total, 19 thanatosis displays were recorded, all from the “Lowlands” sites (5.3% of all captures)—two from Pastrina (a male and a female) and 17 from Slivata (12 males, five females). Overall, sex did not appear to influence thanatosis displays (β = 0.99, 95% CI [−0.03, 2.12], χ^2^ = 3.64, *p* = 0.06). Nevertheless, to account for sexual-size dimorphism, models were evaluated independently for females and males.

**Figure 2:**
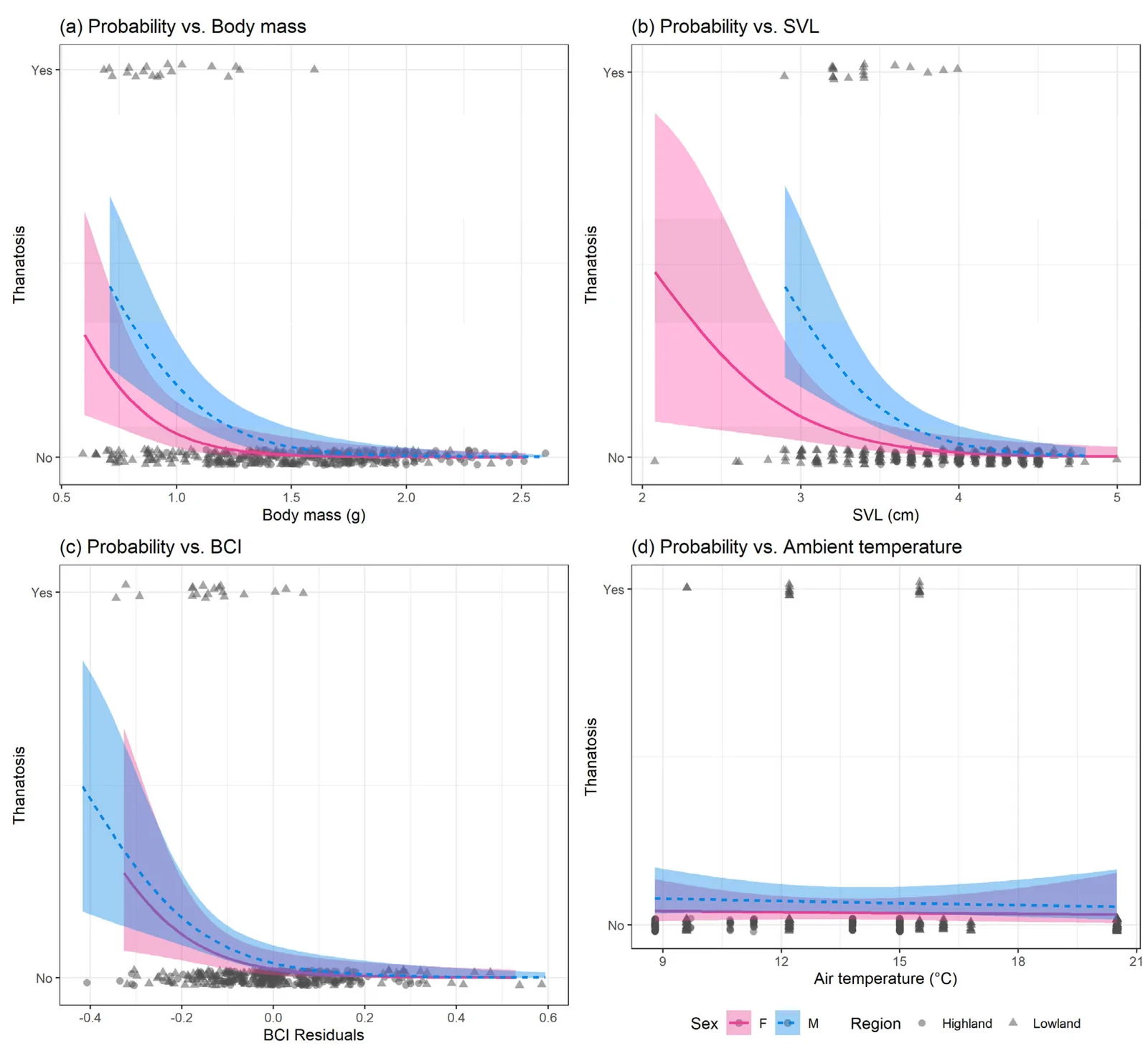
Probability of tonic immobility across four predictors: Body mass (**a**), SVL (**b**), BCI (**c**) and Air temperature (**d**). Distinct regression curves represent predicted probabilities derived from Firth’s penalized bias-reduction logistic regressions, calculated for females (pink lines/ribbons) and males (blue lines/ribbons), bounded by their respective 95% confidence intervals. Individual field observations are jittered slightly along the axes to prevent overlap, with circles representing highland populations and triangles representing lowland populations.

For females, body mass was a highly significant negative predictor of thanatosis, with smaller, lighter females displaying a significantly higher probability of exhibiting the behaviour than larger individuals (β = −4.39, 95% CI [−8.17, −1.69], χ^2^ = 12.25, *p* < 0.001) (Figure 2). This threshold was mirrored by SVL (β = −2.10, 95% CI [−3.85, −0.58], χ^2^ = 7.31, *p* < 0.01) and BCI (β = −8.00, 95% CI [−14.90, −1.97], χ^2^ = 6.99, *p* < 0.01), which also acted as strong negative predictors. Ambient air temperature at the time of collection did not significantly alter the probability of thanatosis display (β = −0.02, 95% CI [−0.24, 0.19], χ^2^ = 0.03, *p* = 0.86).

For males, the pattern was even more pronounced. Body mass again demonstrated a strong negative association with thanatosis (β = −4.03, 95% CI [−6.31, −2.23], χ^2^ = 24.07, *p* < 0.001) (Figure 2), suggesting that smaller male individuals could be predisposed to using this passive defence strategy. SVL (β = −2.68, 95% CI [−4.20, −1.38], χ^2^ = 17.41, *p* < 0.001) followed an identical highly significant trend, and BCI was again significantly linked to expression of the behaviour (β = −7.41, 95% CI [−12.52, −2.83], χ^2^ = 10.44, *p* < 0.001). Again, ambient air temperature had no observable effect (β = −0.03, 95% CI [−0.19, 0.12], χ^2^ = 0.14, *p* = 0.71).

To verify whether the observed negative associations between physical traits and thanatosis were confounded by local population characteristics, a sensitivity analysis using Firth’s penalized logistic regression was performed. It was restricted strictly to the Slivata population. The analysis revealed that body mass (β = −0.60, 95% CI [−3.04, 1.35], χ^2^ = 0.32, *p* = 0.57) and SVL (β = −0.57, 95% CI [−0.98, 2.21], χ^2^ = 0.51, *p* = 0.47) were no longer statistically significant, but BCI remained a negative predictor (β = −5.49, CI [−10.71, −1.01], χ^2^ = 5.99, *p* < 0.05).

## 4. Discussion

Our results indicate that for both sexes, smaller and lighter newts are more likely to display tonic immobility than larger and heavier ones. Body condition was strongly statistically significant for both sexes, and was the only significant predictor when accounting for site-level thanatosis clustering.

Ambient air temperature had no observable effect on the behaviour in either sex, which is in line with previous results on tonic immobility independence from body temperature in Smooth newt juveniles [1], although we have to stress that air temperature at trap collection is not equivalent to body temperature. In the same study, tonic immobility duration was found to be negatively correlated to newt body temperature; unfortunately, working and recording the behaviour in the field (as opposed to the controlled laboratory conditions in [1]), in this study we were unable to reliably measure thanatosis duration. Future field studies could benefit from emerging methodologies—such as automated camera traps and computer vision tools—to monitor thermoregulation and subtle antipredator behaviours in wild ectotherms without human intervention [24]. To our knowledge, the only other published report of thanatosis from field observation of this species is by Kupfer and Teunis, who conducted a long-term study in an area near Bonn, Germany, and reported that 1% of Smooth and Alpine newts caught in their traps demonstrated antipredator behaviour [25]. Although at first this percentage appears much lower than what is reported in this study, it has to be noted that Kupfer and Teunis had accumulated a sample size of over 6000 Smooth newts over the years, and it is likely that our percentage could also change with increasing numbers. More importantly, the authors’ interpretation of “antipredator behaviour” differs from what is accepted in this study, as what they report is essentially “shrinking” behaviour (for definitions, see the Introduction and [12]). In this regard, we think that the present report is the first to document a case of true thanatosis in wild populations of the Smooth newt. Interestingly, we also did not record any shrinking behaviour—although this could be at least partially due to the relatively high temperatures during the study period (i.e., well above the 5 °C temperature threshold for newt activity, following [26]). Kupfer & Teunis [25] also based their observations on animals from terrestrial traps during migration, i.e., a period of relatively low air temperature, when newts had to stay exposed to the air throughout the night. Understanding how environmental temperature influences ectotherm behaviour is particularly important as thermal extremes increasingly alter physiological thresholds and performance across taxa [27]. Kupfer & Teunis [25] consider shrews and carabids caught in their traps to be a major contributing factor to the observed antipredator behaviour. In our case, predators were not present in the buckets where newts were kept after collection, and the behaviour was only displayed during the process of taking measurements or photographs before their release.

The tonic immobility of Smooth newts from this study has all the major elements of true feigning death behaviour, as described in anurans from wild populations [12] and Smooth newts in captivity [1]. It has to be noted that in their experiments, Baškiera et al. [1] intentionally put the juveniles on their backs and recorded the thanatosis duration from that moment (i.e., the behaviour was experimentally induced rather than produced naturally). Similarly to what is reported for anurans [10,28], at the end of the thanatosis, the animals were quick on their feet and trying to escape. Some authors suggest a trade-off between escape ability and thanatosis in ectothermic animals [5], especially at low temperatures [4]; however, these studies were conducted on invertebrate species, and the extent (if any) to which their conclusions could relate to amphibians is unclear. Smooth newts move slowly and high temperatures have lesser effect on their maximum speed compared to other newt species [29], which may predispose them to displays of thanatosis.

To our knowledge, this is the first study that examines the relationship between body condition and displays of tonic immobility in caudate amphibians. Although BCI results for females were only marginally significant, we still think that there is a strong indication that newts with lower relative body condition are more likely to display thanatosis. A meta-analysis by Macdonald et al. [30] found that drought, invasive species and agriculture had pronounced negative effects on body condition in the studied 137 amphibian and reptile species. In this regard, we could hypothesize that the two populations where this behaviour was observed offer worse conditions compared to the others. Both of these populations were at low altitudes and it may be tempting to connect altitude with thanatosis and body condition. It is, however, worth noting that while newts from Yarlovo were largest and heaviest, their BCI was not statistically different to that of newts from Pastrina, one of the lowlands sites. Even more tellingly, females from Pastrina did not differ from females from Parshevitsa (a highland site) in neither body mass, SVL, nor BCI. In their study on *Salamandra salamandra* (Linnaeus, 1758), Schulte & Caspers [11] found no difference in tonic immobility displays between larvae from pond and spring habitats, suggesting that habitat characteristics are less important for this type of behaviour. Interestingly, these authors also found no effect of size on the amount of time the tonic immobility behaviour was displayed by the larvae. This is in contrast to our results, where size and body mass were invariably good predictors of the behaviour. Results in this regard appear to be mixed, as according to Oswald et al. [31], larger fire salamander larvae from both pond and stream habitats were more risk-prone than smaller ones. It is possible that the relationship is more pronounced in adults, but without additional data, such a statement would be too speculative. For adults of the larger species *Triturus ivanbureschi*, Lukanov & Atanasova [32] established that individual BCI scores were correlated with individual activity (i.e., newts with higher BCI scores were recaptured more often), and this relationship was independent of both sex and temperature. In this regard, it could be hypothesized that newts with lower BCI scores would be less likely to move and, consequently, more likely to use immobility as a defence strategy. Unfortunately, in the present study we were unable to conduct individual recognition of captured animals, as the ventral pattern in many newts was too generic (i.e., too few spots that do not form a reliably recognizable pattern).

## 5. Conclusions

In conclusion, our field study provides the first empirical evidence of true tonic immobility in wild populations of the Smooth newt, demonstrating that this passive antipredator response is strongly associated with individual physical state rather than sex or ambient air temperature. There was a significant inverse association between thanatosis display probability and overall body scale (body mass, SVL, and BCI). The sensitivity analysis at the site where displays were most frequently recorded (Slivata) demonstrated that relative body condition (BCI) remained a strong predictor, while body mass and SVL lost significance. This indicates that lower body condition index, rather than absolute size or weight, could be important for death-feigning in wild populations. Although the sensitivity analysis controls for spatial clustering, the occurrences remained concentrated across two specific field visits, meaning temporal/sampling-event context remains a potential co-factor, and highlighting the need for multi-population studies tracking individual behavioural consistency over time. Future research measuring thanatosis duration across broader geographical and altitudinal gradients will be essential to further resolve how environmental factors and energetic constraints interact to shape antipredator strategies in caudate amphibians.

## Figures and Tables

**Table 1:** SVL and body mass of captured newts from the four study populations. Data are presented as Mean ± SD (Min–Max). Statistically significant differences (*p* < 0.01) between males and females from each population are marked with an * next to the higher value.

Site	Males			Females		
	SVL (cm)	Body Mass (g)	n	SVL (cm)	Body Mass (g)	n
Pastrina	* 4.12 ± 0.35 (3.10–4.80)	* 1.64 ± 0.26 (1.22–2.37)	52	3.73 ± 0.42 (2.90–5.00)	1.37 ± 0.24 (0.97–2.10)	48
Parshevitsa	* 3.95 ± 0.23 (3.40–4.30)	1.34 ± 0.23 (0.72–1.74)	18	3.71 ± 0.29 (3.20–4.40)	1.32 ± 0.32 (0.71–1.95)	18
Slivata	* 3.41 ± 0.29 (2.90–4.20)	1.03 ± 0.27 (0.71–2.03)	45	3.16 ± 0.36 (2.08–4.10)	0.89 ± 0.24 (0.60–1.73)	32
Yarlovo	* 4.25 ± 0.23 (3.70–4.70)	1.83 ± 0.28 (1.23–2.60)	77	4.11 ± 0.27 (3.50–4.60)	1.78 ± 0.32 (1.22–2.52)	67

**Table 2:** Tukey HSD comparison of SVL, body mass and BCI across sites, separated by sex. Only cases with statistically significant differences (*p* < 0.01) are shown.

	Pastrina	Parshevitsa	Slivata	Yarlovo
	Males			
Pastrina	-	Body mass, BCI	SVL, Body mass	Body mass
Parshevitsa	Body mass, BCI	-	SVL, Body mass	SVL, Body mass, BCI
Slivata	SVL, Body mass	SVL, Body mass	-	SVL, Body mass, BCI
Yarlovo	Body mass	SVL, Body mass, BCI	SVL, Body mass, BCI	-
	Females			
Pastrina	-		SVL, Body mass	SVL, Body mass
Parshevitsa		-	SVL, Body mass	SVL, Body mass
Slivata	SVL, Body mass	SVL, Body mass	-	SVL, Body mass, BCI
Yarlovo	SVL, Body mass	SVL, Body mass	SVL, Body mass, BCI	-

## Data Availability

The original contributions presented in this study are included in the article/Supplementary Material. Further inquiries can be directed to the corresponding author.

## Supplementary Materials

The following supporting information can be downloaded at https://www.mdpi.com/article/10.3390/ani16162616/s1, Table S1: raw data used for the analyses; Video S1: display of thanatosis in a female Smooth newt from Pastrina; Figure S1: photographic setup used for recording ventral patterns of captured Smooth newts (16 March 2026).

## Abbreviations

The following abbreviations are used in this manuscript:

BCI	Body condition index
SVL	Snout-vent length
